# Direct Regulation of Striated Muscle Myosins by Nitric Oxide and Endogenous Nitrosothiols

**DOI:** 10.1371/journal.pone.0011209

**Published:** 2010-06-18

**Authors:** Alicia M. Evangelista, Vijay S. Rao, Ashley R. Filo, Nadzeya V. Marozkina, Allan Doctor, David R. Jones, Benjamin Gaston, William H. Guilford

**Affiliations:** 1 Department of Biomedical Engineering, University of Virginia, Charlottesville, Virginia, United States of America; 2 Department of Pediatrics, University of Virginia, Charlottesville, Virginia, United States of America; 3 Thoracic and Cardiovascular Surgery, University of Virginia, Charlottesville, Virginia, United States of America; 4 Department of Pediatrics, Washington University, St. Louis, Missouri, United States of America; Ohio State University, United States of America

## Abstract

**Background:**

Nitric oxide (NO) has long been recognized to affect muscle contraction [1], both through activation of guanylyl cyclase and through modification of cysteines in proteins to yield S-nitrosothiols. While NO affects the contractile apparatus directly, the identities of the target myofibrillar proteins remain unknown. Here we report that nitrogen oxides directly regulate striated muscle myosins.

**Principal Findings:**

Exposure of skeletal and cardiac myosins to physiological concentrations of nitrogen oxides, including the endogenous nitrosothiol S-nitroso-L-cysteine, reduced the velocity of actin filaments over myosin in a dose-dependent and oxygen-dependent manner, caused a doubling of force as measured in a laser trap transducer, and caused S-nitrosylation of cysteines in the myosin heavy chain. These biomechanical effects were not observed in response to S-nitroso-D-cysteine, demonstrating specificity for the naturally occurring isomer. Both myosin heavy chain isoforms in rats and cardiac myosin heavy chain from human were S-nitrosylated *in vivo*.

**Significance:**

These data show that nitrosylation signaling acts as a molecular “gear shift” for myosin—an altogether novel mechanism by which striated muscle and cellular biomechanics may be regulated.

## Introduction

Products of nitric oxide (NO) synthase activation have long been recognized to affect muscle contraction [1]. Contraction and contractility are thought to be regulated indirectly through activation of guanylyl cyclase to generate cGMP, and by direct action of NO on proteins. Protein sulfhydryl modification by oxidized NO to yield S-nitrosothiol (S-NO) moieties is widely accepted as an important regulatory mechanism. The breadth and importance of the protein targets, however, remains to be fully understood.

Skinned (membrane-permeablized) muscle preparations respond to NO donors with reduced Ca^2+^ sensitivity [2]–[4], ATPase activity [2], [3], and velocity of shortening [3]. In intact cardiac fibers NO causes a decrease in calcium sensitivity and an increase in rigor force [5]. These data suggest a direct effect of NO on myofibrillar proteins in muscles that is independent of calcium handling and cGMP-mediated phosphorylation. Unfortunately, it is difficult to determine in these preparations which of the myofibrillar proteins are being affected and how. It was reported in a 1998 abstract that NO donors completely inhibit myosin function as measured *in vitro* (J.L. Tan, M. Heidecker, J.D. Cohen, M.B. Fowler & J.A. Spudich, 1998). In recent work, Nogueira and coworkers found that the ATPase activity of skeletal myosin can be reversibly inhibited by nitroso-S-glutathione but not by donors of NO [6]. These studies together suggest that myosin is a target for regulation by endogenous donors of NO.

To gain a better understanding of whether and in what manner the physiological function of myosin is affected by NO, we tested the effects of a NO donor (DEA NONOate) and small, endogenous nitrosothiols (nitroso-cysteine) on the force and velocity generated by skeletal and α-cardiac myosins *in vitro*. We found that DEA NONOate reduced actin filament velocity over striated myosins, but increase isometric force generated by myosin. While nitroso-L-cysteine had a similar effect to NONOates, nitroso-D-cysteine had no functional effect on myosin, showing stereospecificity for the endogenous NO donor. Our data show a strong yet nuanced effect of NOS products on striated muscle myosins that extends beyond mere inhibition, suggesting that myosin is indeed directly regulated by NO.

## Results

### NO slows motility by striated myosins and HMM

Using the *in vitro* motility assay we observed the movement of single fluorescently labeled actin filaments gliding over a myosin-coated glass surface [7], [8]. We employed a modified version of the protocol that avoids the use of reducing agents that would otherwise reverse S-nitrosylation [9]. Briefly, myosin or heavy meromyosin (HMM) was bound to a nitrocellulose-coated coverslip in a flow cell and flushed with a reducing agent-free buffer. HMM is a proteolytic subfragment of myosin that includes the paired heads of myosin, the associated light chains, and the short coiled-coil region called S2, but lacks the tail that allows myosin to form thick filaments. DEA NONOate was diluted into buffer pre-equilibrated to either 15 or 152 mmHg PO_2_ and added immediately to the flow cell for 1 minute. Actin filament motion was observed by epifluorescence microscopy after removing the donor.

**Figure 1:**
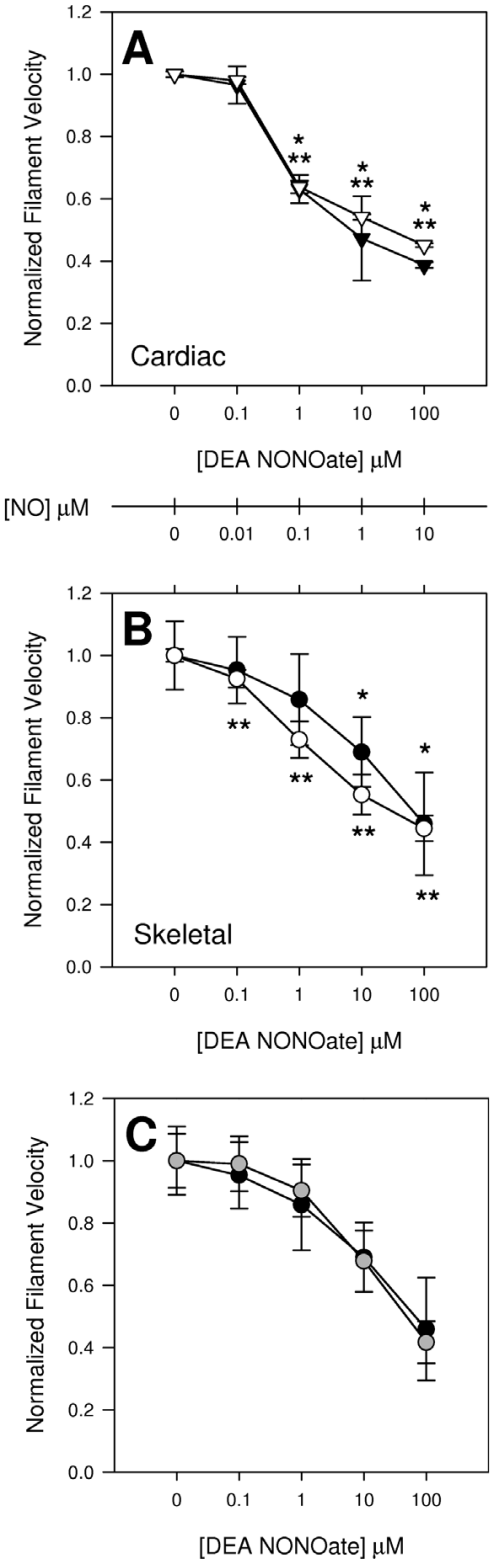
Dose-response and PO_2_ interactions of the NO donor DEA NONOate on myosin. A: Cardiac myosin at high (open symbols, 20%, 152 mmHg) and physiologic (solid symbols, 2%, 15 mmHg) PO_2_. PO_2_ has no significant effect on the response of isolated cardiac myosin to the NONOate. B: Same as A, but with skeletal muscle myosin which was significantly more sensitive to NO at lower PO_2_. *p<0.05 compared to no DEA NONOate at 15 mmHg. ** p<0.05 compared to no DEA NONOate at 152 mmHg. C: There is no difference in the dose-response curves of full length skeletal myosin (gray) and HMM (black), indicating that the site of NO action lies within the head or S2 regions of myosin. N = 3. The [NO] scale bar shows the approximate NO concentration for the equivalent DEA NONOate concentration given our exposure times and conditions.

We found a dose-dependent response of both cardiac and skeletal muscle myosin function to DEA NONOate (Figure 1A and B), though cardiac myosin was more sensitive than skeletal. There was no difference in the response of full-length skeletal myosin and skeletal HMM to donor, indicating that the responsive domain of myosin is in subfragments 1 or 2 (Figure 1C). These effects were reversible by exposure to 10 mM DTT, and could not be duplicated using sulpho NONOate, which is less likely than DEA NONOate to transfer NO^+^ to thiols.

S-nitrosylation by NO radical itself requires an electron acceptor and therefore can be oxygen-dependent. We therefore measured dose-response curves at three oxygen concentrations – 0, 15 (physiological), and 152 (atmospheric) mmHg PO_2_. Cardiac myosin was approximately 10X more sensitive to donor than was skeletal when measured in terms of the concentration to achieve a significant reduction in velocity. Inhibition of motility in cardiac myosin did not differ significantly at physiological and atmospheric PO_2_ (Figure 1A, p = 0.25). The K_i_ for reduction of actin filament velocity propelled by cardiac myosin was 660±250 nM donor, or approximately 66 nM [NO] at 152 mmHg PO_2_. In contrast there was a trend toward blunting of the skeletal muscle myosin dose response at high PO_2_ (Figure 1B); the K_i_ for inhibition was 3240 and 138 nM [NO] at 152 and 15 mmHg PO_2_, respectively (p = 0.03).This suggests that at physiologic oxygen concentrations the response of myosin to nitrogen oxides is already maximized. In the absence of oxygen, 1 mM DEA NONOate had no effect on motility. These data show that myosin function is responsive to NO in an oxygen- and isoform-dependent manner.

### Myosin generates more force in the presence of NO

**Figure 2:**
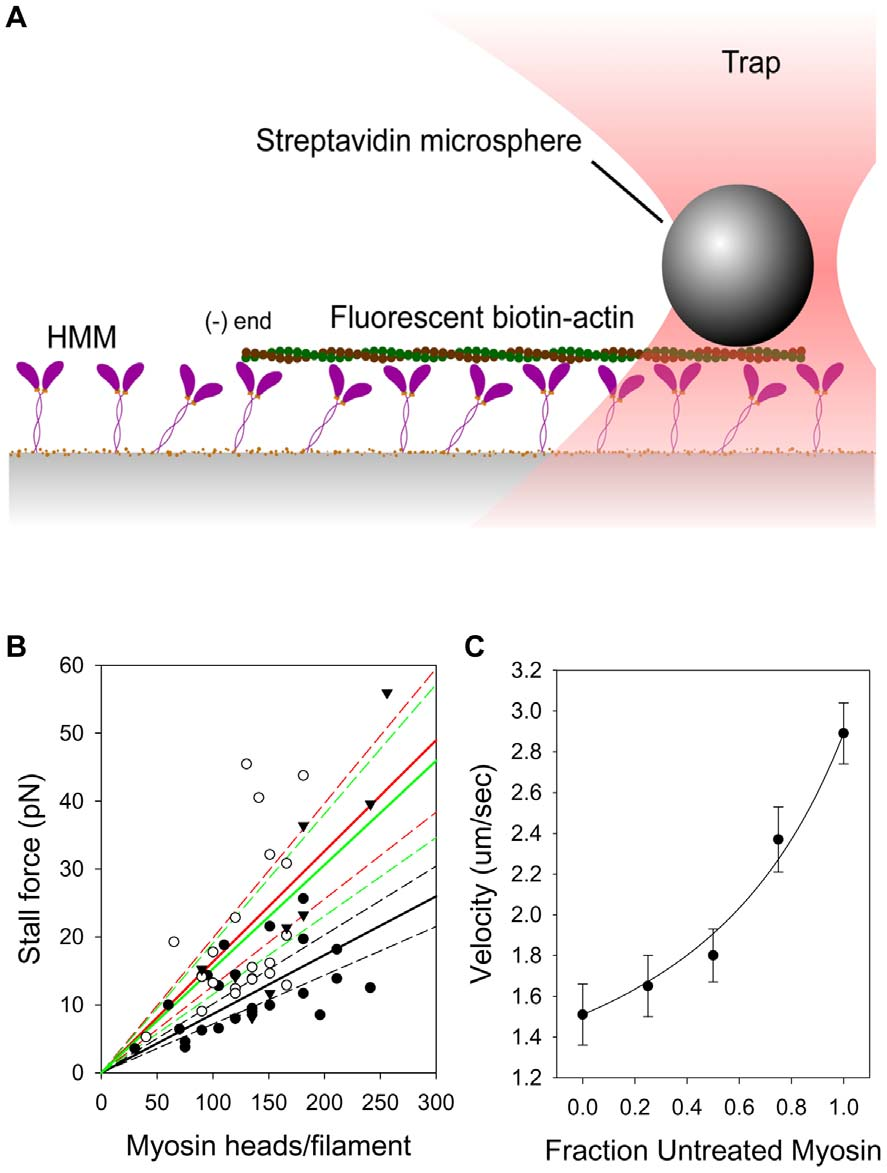
Measurement of time-averaged isometric force after exposure to NO. A: A streptavidin-coated bead is held in a laser trap and touched to the trailing (+) end of a moving, biotinylated, TRITC-phalloidin labeled actin filament. Displacement of the bead from trap center was followed using back focal plane interferometry [34], [35]. B: Force versus number of potentially bound, force-generating heads (a reflection of filament length) for three conditions, control (•, black lines), 10 µM DEA NONOate (○, red lines) and 100 µM DEA NONOate (▾, green lines). Steeper slopes indicate proportionately higher forces per head. Solid lines indicate the regression fit, while the dashed lines show 95% confidence intervals. Each data point is an independent force measurement (N = 25, 20 and 10 for 0, 10, and 100 µM DEA NONOate respectively). C: Mixtures experiment for rat cardiac myosin. The line shows the fit of equation 1 from Harris et al. [11] yielding a relative force production 2.1-fold higher after DEA NONOate-treated compared to control. N = 3.

As a physiologically-relevant measure of the force production in the purified actomyosin system, an optical trap assay was used to measure the stall force of multiple myosin molecules interacting with an actin filament [10]. Laser trapped beads were bound to the trailing ends of motile actin filaments (Figure 2A) to measure the force at which forward motion stalled. Repeated over a range of actin filament lengths, these measurements can be used to estimate the time-averaged isometric force generated by myosin.

Skeletal HMM was treated with DEA NONOate at 0, 10 or 100 µM for 1 minute. Both 10 and 100 µM caused force generation by myosin to nearly double - 1.9±0.2 and 1.8±0.2 fold over control as indicated by the slopes of the force/heads/filament relationship (Figure 2B). To confirm these results, we performed “mixture” experiments to measure the relative force generated by nitrosylated myosins. Analogous to a tug-of-war, donor-treated and untreated myosins are laid down in a motility assay over a range of relative concentrations, ranging from 100% treated and 0% untreated, to 0% treated and 100% untreated. The relationship between filament velocity and fractional composition can be used as a measure of relative force production by the two myosin populations [11]. At any given mixture, velocities of actin filament velocities were closer to those of NO^+^ -treated myosin than to control myosin (Figure 2C). Interpreted according to Harris et al. [11], the data yield a relative production of 2.1±0.3 higher force by DEA NONOate-treated cardiac myosin compared to untreated myosin (p = 0.003). These data too suggest that NO (as NO^+^) exposure approximately doubles the time-averaged force generated by skeletal and cardiac myosins.

### Myosin is stereoselective for an endogenous NO donor

**Figure 3:**
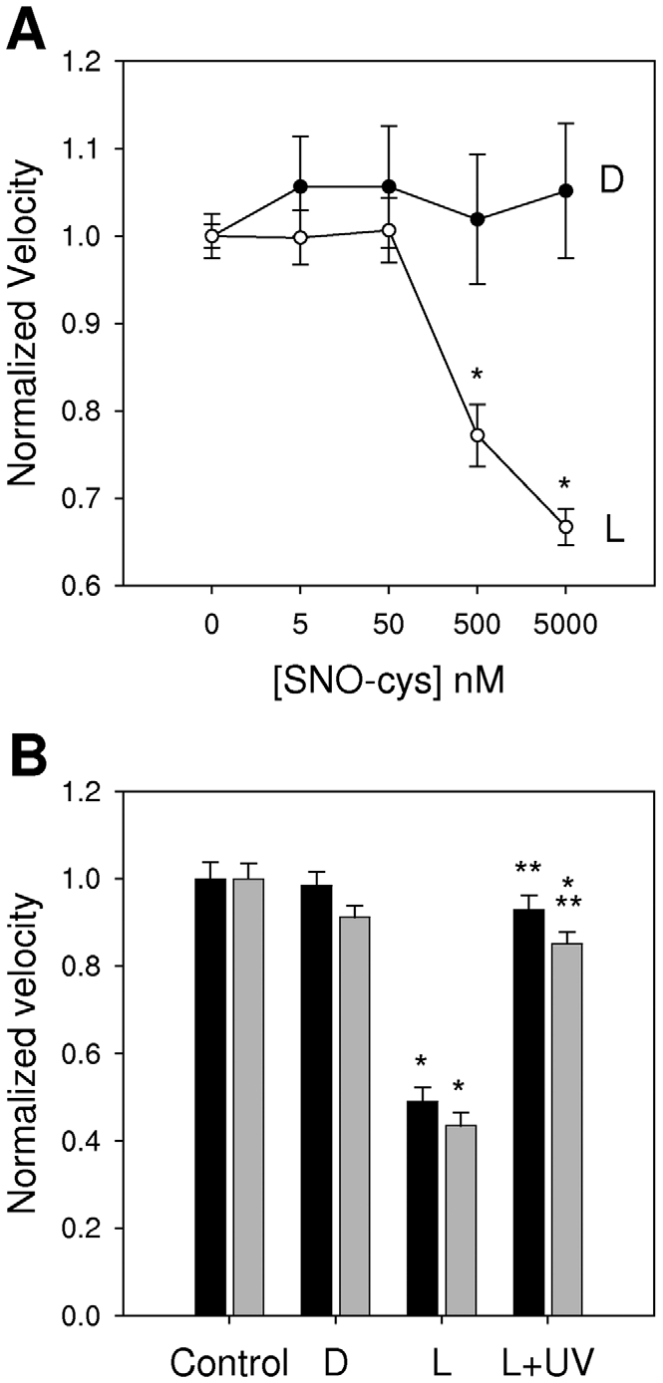
Stereo-selective effects of SNO-L-cysteine on myosin. A: Dose-response showing that SNO-L-cysteine, the naturally occurring isomer, has pronounced effects on actin filament velocity, while SNO-D-cys does not. B: Effects of 5 µM L- and D-isomers of SNO-cys on actin filament velocities over skeletal (dark bars) and cardiac (light bars) myosin, and recovery by exposure to ultraviolet light – strong support of nitrosylation as the underlying modification. *Different from control (p<0.05). **Different from SNO-L-cys alone (p<0.05). N = 5.

An effect of exogenous nitrogen oxide on myosin function does not necessarily imply regulation. We therefore used the naturally occurring donor nitrosocysteine (SNO-cys) - a nitrosothiol in cells that may be important in signaling. Its D- and L-isomers have similar chemistries and rates of decay to yield NO^+^, so any preference in the response to the naturally occurring L-isomer over the synthetic D-isomer is suggestive of stereospecific regulation via trans-nitrosylation – the transfer of NO^+^ from a nitrosothiol to a thiol. To test for functional stereospecificity to an endogenous donor, skeletal and cardiac myosins were exposed for 1 minute in the dark to SNO-L-cys and SNO-D-cys. We found that SNO-D-cys had no effect on myosin-based motility (Figure 3A). In contrast, SNO-L-cys reduced actin filament velocities by approximately one half at 5 µM. Motility was restored to near control levels by two 2-minute exposures to ultraviolet light (Figure 3B) which breaks S-NO bonds [12] with buffer washes after each exposure to remove the freed NO. Stereo-selectivity for the naturally occurring isomer of a small ligand is generally considered strong evidence for specificity, and hence regulation.

The effects of SNO-cysteine and other nitrogen oxides on myosin are unlike those of alkylating agents (e.g. N-ethylmaleimide), which completely inhibit myosin cycling and lead to the formation of strong, rigor-like bonds with actin.

### Myosin heavy and light chains are nitrosylated *in vitro* and *in vivo*

We next tested whether the L- or D-isomers of SNO-cysteine could nitrosylate myosin. Skeletal muscle HMM was exposed at a 1∶2 molar ratio with each isomer (12 µM) and the incorporated NO measured by chemiluminesence. We found that SNO-L-cys lead to the incorporation of 1.16 NO-equivalents/myosin heavy chain, while SNO-D-cys lead to the incorporation of only 0.25. These data suggest that myosin is more efficiently nitrosylated by the L- than by the D-isomer of S-NO-cys. Liberation of NO by SNO-D-cys during removal by microdialysis may explain the small level of stereoselectivity; the nitrosylation state at the earliest time points after SNO-cys exposure remains to be determined. Nonetheless, nitrosylation of myosin is stereo-selective and while reversible, persists over time.

To determine which chains of the myosin heterohexamer were stereospecifically nitrosylated, we used a coumarin switch assay [13], similar to the standard biotin switch assay [14] to identify nitrosylated proteins in a mixture. In this variation, AMCA fluorophore rather than biotin replaced all NO moieties so that nitrosylated proteins could be rapidly imaged under UV light and subsequently stained with colloidal coomassie to determine the mass for each protein band. Normalizing the nitrosylation signal to total protein is critical for quantifying nitrosylation in myosin heavy chain, or for any other protein where (a) sample-to-sample protein recovery after the biotin switch is not uniform, or (b) the number of modified cysteines is low relative to the mass of the protein. Both are the case for myosin heavy chain and failure to normalize against the mass of protein in each individual electrophoretic band (as opposed to an overall protein assay) will mask a small number of consistently nitrosylated cysteines in the large protein.

**Figure 4:**
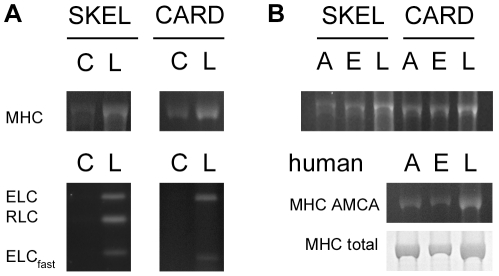
Myosin nitrosylated *in vivo* and *in vitro*. A: Rat skeletal and cardiac myosin heavy (MHC) and light chains were nitrosylated by *in vitro* exposure to SNO-L-cysteine (L) over the control level (C). The ventricular/slow skeletal isoform of the essential light chain (ELC) could be nitrosylated, as could the regulatory light chain (RLC) and fast essential light chain of skeletal muscle myosin. The regulatory light chain of cardiac myosin was not significantly nitrosylated. B: Myosin heavy chain was endogenously (E) nitrosylated in rat skeletal and cardiac muscle, and also in human myocardium (MHC AMCA). A protein staining of the same gel is shown (MHC total) to illustrate how nitrosylation is normalized against protein mass to reveal low levels of nitrosylation between zero (A, ascorbate-treated) and maximum (L, SNO-L-cys-treated). See the text for details of the normalization procedure. Without normalization, variations in recovery between protein bands (as shown, typical) will mask single (or a few) nitrosylated cysteines in large proteins like myosin. N = 3 in all cases.

All three isoforms of myosin heavy chain and most of their associated myosin light chains were readily nitrosylated by SNO-L-cys (Figure 4A) – these included the slow and fast isoforms of the essential light chains, and the skeletal regulatory light chain. The only light chain that was not significantly nitrosylated by SNO-L-cys was the cardiac myosin regulatory light chain. The myosin heavy chain was significantly stereoselective for nitrosylation by the L- over the D-isoforms of SNO-cys as assayed by coumarin switch. The L/D nitrosylation per unit mass was 1.9±0.1 and 3.8±0.9 for skeletal and cardiac myosin heavy chains, respectively (p<0.001 for a ratio >1.0). Once again, the low degree of stereoselectivity was probably related to the extended times necessary to remove the protein from donor using precipitating agents. We found no significant selectivity by any of the light chains for nitrosylation by L- over D- SNO-cys (L/D nitrosylation per unit mass ∼1). This suggests that the functional stereoselectivity we observed (Figure 3) is the result of heavy chain nitrosylation, and not light chain nitrosylation.

We also measured the endogenous level of nitrosylation in rat skeletal and cardiac myosins, and human cardiac myosin. High-ionic strength extracts of these muscles were left untreated (endogenous), treated with ascorbate (control), or treated with 10 µM SNO-L-cys (L) and subjected to the coumarin switch assay followed by coomassie staining and protein mass normalization as described earlier. Relative endogenous nitrosylation was calculated after mass normalization as (endogenous - control)/(L - control). We found that rat skeletal and cardiac myosin heavy chains were nitrosylated *in vivo* to 50% and 30%, respectively, of the level induced using 10 µM SNO-L-cys. Human cardiac myosin heavy chain was nitrosylated to a level of approximately 20% (Figure 4B). These are minimum estimates, since there will be some spontaneous loss of NO equivalents during protein extraction. We were not able to make comparable measurements of light chain nitrosylation since the high ionic strength extracts had an abundance of low molecular weight proteins that could lead to misidentification of the light chain bands.

## Discussion

Our data suggest that either nitrosylation or trans-nitrosylation act as a “gear shift” for myosin, switching it on-the-fly from a relatively high-speed, low-force motor to a low-speed, high-force motor at physiological concentrations of NO. The question naturally arises, to what purpose? Regulation of contraction under physiological and pathological conditions by NO is complex, involving direct and cGMP-dependent pathways [15], so a simple answer is unlikely to suffice. It has been proposed that the direct effect of NO in muscle cells is to slow contraction and its associated metabolism [16]. One possible example of this is exercise, during which NO increases in skeletal muscle [17]. NO or S-nitrosothiols may thus serve both to increase oxygen supply to the tissue through vascular dilation and simultaneously shift contractile function to higher force generation at the expense of lowered shortening velocity.

The concentrations of S-nitrosothiols used in these experiments are thought to span the physiological range. Free [NO] on the order of 100 nM to several µM has been measured adjacent to stimulated cardiac myocytes [18]–[21]. However, the presence of these levels of free radical in the presence of ∼200 µM concentrations of myoglobin in the myoplasm seems unlikely [22]. It is entirely possible that the concentrations of nitrosothiols in myocytes and other cells are ∼100 nM [23], [24]. However, as with any reversible bimolecular reaction, it is not only the concentration of reactants but the forward and reverse reaction rate constants for the nitrosylation reaction that will ultimately determine the functional impact of NO production. The forward reaction we know from motility experiments occurs in seconds. We also know that the effects of NO donors persist for at least several minutes once donor is removed, presumably due to slow spontaneous reversal of the S-NO modification. Thus S-NO-myofibrillar proteins may accumulate in cells, even in the presence of myoglobin.

The pattern of effects on force and actin filament velocity suggests a model where S-NO modification of muscle myosin alters the attached time and duty cycle of myosin. From the perspective of a single molecule, the velocity of actin over a pure myosin is related to the inverse of its attached lifetime (t_on_) – how long during each hydrolytic cycle myosin remains attached to actin. In contrast, the time-averaged force generated by myosin is related to its duty cycle (t_on_/(t_on_+t_off_)) – the fraction of the total cycle time myosin is attached to actin. Thus a doubling of t_on_ with no change in the detached time (t_off_) would result in a 50% decrease in actin filament velocity, and an approximate 2X increase in average force. Striated muscle myosins are thought to have low duty cycles; thus doubling t_on_ could produce the observed changes in force and velocity without large changes in total cycle time as measured by ATPase rates. This may explain why Nogueira and coworkers [6] found no effect of DEA NONOate on ATPase rates; in addition, in those particular experiments their readout of myosin function was a non-functional, ion-activated ATPase assay rather than a functional actin-activated assay.

One might speculate that nitrosylation is a general regulatory mechanism for myosin-based motility. There are a number of cysteines that are well conserved across myosin isoforms, including two especially reactive cysteines [25] (cys707 and cys697) among the nine in the motor domain of the heavy chain. Interestingly, the reactive cysteines themselves and the encompassing alpha helix are highly conserved in muscle myosins, even across species. However, conservation of the reactive cysteines does not fully extend to non-muscle myosins. Myosin V, for example, possesses one of the two reactive cysteines and conserves much of the encompassing helix from muscle myosins while other myosins have neither reactive cysteine. If one or both reactive cysteines are indeed the point of NO regulation of muscle myosin, then one would predict that myosins lacking these cysteines would be unresponsive to NO and nitrosothiols. It is possible, however, that non-muscle myosins incorporate different sites for regulation by NO, including one proposed in myosin heavy chain 9 [26], that better meet their particular regulatory needs. Further, we found that some of the light chains of striated myosins can be nitrosylated *in vitro*. There are obviously several possible sites for regulation of myosins by NO and its endogenous donors. Determining which of these is responsible for the effects observed here will be the subject of future studies, as will the identification of other potential NO regulatory sites in the contractile apparatus of cells.

## Methods

### Ethics Statement

Use of human myocardium was approved by the University of Virginia Human Investigation Committee, Protocol 10274 to Drs Jones and Gaston to use “materials (data, documents, records or specimens) that have been collected solely for non-research purposes (such as medical treatment and/or diagnosis).” Rat myocardial and skeletal muscle tissues were obtained from the cadavers of animals sacrificed under other investigators' IACUC protocols; no animals were sacrificed specifically for this study, and it was therefore deemed exempt from IACUC approval.

### Proteins

Myosin was prepared from rat skeletal and cardiac tissues by the method of Shiverick [27] and actin according to Pardee and Spudich [28]. Skeletal HMM was prepared from fresh myosin according to Margossian and Lowey [29]. Actin was biotinylated as described previously [10].

### *In vitro* motility assays and donor treatments

Exposures to NO donors take place in reducing agent-free solutions bubbled with N_2_/O_2_ to obtain the desired oxygen tension. NONOates (DEA and sulpho) were obtained commercially (Alexis/Cayman) and stored frozen in 100 µM KOH. Stock NONOates were diluted in 10 µM KOH <30 seconds before use. Treatment buffers were formulated such that the pH of the reaction mixture changes by less than 0.1 units upon addition of donor. L- and D-S-NO-cys were prepared as 10 mM stock solutions and were stored as aliquot at −80°C. Stock SNO-cys was diluted to working concentrations in actin buffer <30 seconds before use.

The DTT-free motility assay is described elsewhere [9]. Briefly, aliquots of all solutions were vacuum degassed on ice for 20 minutes. Preparations were performed in a darkened room to prevent donor photolysis. Myosin or HMM was applied to a flow cell consisting of a nitrocellulose-coated coverslip and glass slide to a final concentration of 200 µg/ml and 80 µg/ml, respectively. The flow cell was washed with degassed “actin buffer” (25 mM KCl, 25 mM Imidazole, 1 mM EGTA, 4 mM MgCl_2_, pH 7.4) after a one minute incubation. Donor was applied to the flow cell in actin buffer equilibrated with 0%, 2%, or 20% oxygen (balance nitrogen). After a one minute incubation with donor, the flow cell was washed with degassed low salt buffer and blocked with 0.5% (v/v) Tween 20 in degassed low salt buffer for myosin, or 2% PVP40 in degassed low salt buffer for HMM for 1 min. TRITC-phalloidin labeled actin filaments were introduced, followed by two degassed actin buffer washes. A low salt motility buffer (25 mM KCl, 25 mM Imidazole, 1 mM EGTA, 4 mM MgCl_2_, 1 mM ATP, 0.5% methylcellulose, pH 7.4) was added and actin filament movement was recorded. The motility buffer also contained an oxygen scavenger system described in Guo and Guilford [30].

Actin filaments were tracked used a energy-minimalization segmentation routine, previously described [9]. At least 50 actin filaments were tracked for each experimental trial, though typically hundreds were used, and each condition repeated a minimum of three times. The sample number (N) was conservatively taken as the number of independent experiments, not the number of filaments tracked. Filament velocities were normalized to daily controls.

### Laser trap force assay

As a physiologically-relevant measure of the force production in the purified actomyosin system, an optical trap assay was used to measure the stall force of multiple myosin molecules interacting with an actin filament [10]. Briefly, 0.97 µm streptavidin-coated microspheres (Bangs Laboratories) were fluorescently labeled with TRITC-labeled BSA. HMM was applied to flow cells and subsequently blocked with 1 mg/ml BSA. TRITC-phalloidin labeled and biotinylated actin filaments were added. Finally, streptavidin microspheres were resuspended in actin buffer with 100 µM ATP and introduced to the flow cell to initiate motility. Individual fluorescent beads were trapped and a motile actin filament was chosen. A trapped bead was brought into contact with the trailing (+) end of the motile filament and held until the forward motion of the filament stalled. Stall force measurements were repeated over a range of actin filaments lengths (1–7 µm). The number of HMM heads that may interact with each actin filament was estimated from HMM density measurements [30].

### Mixture Assay

Force generation by donor treated myosin relative to untreated myosin was measured using a mixture assay [31], [32] with modifications. Myosin was exposed in solution to DEA NONOate or nitrosocysteine in buffer bubbled with 2% or 20% oxygen and allowed to sit for 35 minutes on ice. Treated and untreated myosin were mixed in ratios of 100∶0, 75∶25, 50∶50, 25∶75 and 0∶100 immediately before application to a flow cell. Motility was then performed as described above. An alternative approach was also used, namely introducing 50% diluted myosin to the flow cell, treating with donor for one minute (as above), and subsequently introducing a second bolus of 50% diluted myosin. The two methods gave comparable results.

Mixture assay data were analyzed by the model of Harris et al. [11] assuming a compression factor of 0.26. The model was fit to the data using SigmaPlot to find the force of the fast myosin relative to the slow.

### Nitrosothiol quantitation

S-nitrosothiols were measured in 6 µM skeletal HMM after treatment with 12 µM L- or D-SNO-cysteine, and with or without HgCl_2_ pretreatment to break the S-nitrosothiol bond (negative control). Samples were dialyzed for 1.5 hours to remove excess SNO-cysteine. Assays are carried out under He in a purge vessel containing 1 mM cysteine and 100 µM CuCl, pH 7.0, 50°C connected to an NOA chemiluminescence detector (Sievers NOA 280, Boulder, CO) with added carbon monoxide (CO) to the inert gas flow through the reflux chamber, preventing NO autocapture by heme groups. Metal carbonyls (≈0.7 ppm in research grade CO) must be removed, as both Ni- and Fe-carbonyls chemiluminescence in the presence of O_3_. Therefore, the CO source gas is passed through iodine crystals and activated charcoal, blended with the He stream in a gas proportioner. Oxidized cys was replaced, and residual Hb removed, by refreshing the reflux chamber after each sample injection [33]. Water and nitrite NO_2_^−^ solution were injected as additional negative controls before each experiment.

### Coumarin switch assay

S-nitrosylation was measured using a modification [13] of Jaffrey's biotin switch assay [14]. AMCA-HPDP was used rather than biotin-HPDP during labeling to allow S-nitrosylated proteins to be imaged in-gel prior to staining total protein; this aids in normalization of fluorescence against protein mass in each band. Between treatments, protein was precipitated using either 10% trichloroacetic acid (TCA) at room temperature for 10 minutes, or 90% cold acetone (−20°C) for 20 minutes followed by 10 minutes of centrifugation, depending on whether the myosin heavy chain or myosin light chains, respectively, were desired; myosin heavy chain does not precipitate constitutively in acetone, and myosin light chains do not precipitate constitutively in TCA.

Purified skeletal or cardiac myosin in HEN buffer (HEPES, EDTA, neocuproine, as in [14]) were exposed to SNO-cys. After precipitation to remove donor, reduced cysteines were blocked with 1% methyl methanethiosulfonate (MMTS) in HENS (HEN with 2% SDS) at 50°C for 30 minutes. Protein was precipitated twice to remove MMTS, and resuspended in 1 mM AMCA-HPDP in HENS with 4 mM sodium ascorbate. Labeling was at room temperature in the dark for 1 hour. Protein was precipitated to remove excess label, and resolved on 6% (for myosin heavy chain) or 12% gels (for myosin light chains). ACMA-labeled protein was imaged in an Alpha Innotech gel documentation system using a 460 nm filter and an excitation wavelength of 365nm. Gels were subsequently stained with colloidal coomassie and imaged in brightfield for total protein. The identity of the ventricular/slow skeletal isoform of the essential light chain band was confirmed using Western blot against MYL3.

### Statistics

Statistical comparison of means was by z-test. The slopes of fitted lines, such as those from the laser trap assay, were compared as described in Rao and coworkers [10]. N unless otherwise stated is the number of independent experimental preparations, not the number of individual samples.
